# Book Notices

**Published:** 1870-09

**Authors:** 


### Book Notices.

The Physiology of Man; Designed to represent the existing state of Physiological Science, as applied to the functions of the Human Body, By Austin Flint, Jr., M.D., Professor of Physiology and Microscopy, in the Bellevue Hospital Medical College, New York; Fellow of the New York Academy of Medicine; Microscopist to Bellevue Hospital. New York: D. Appleton & Co., 90, 92, and 94 Grand Street. 1870. Price, $4.50.

This is the third volume of Dr. Flint's valuable work on the Physiology of Man; and, like the two preceding volumes, it is published on excellent type and paper. It contains about 530 pages, and embraces the consideration of the functions of Secretion; Excretion; Ductless Glands; Nutrition; Animal Heat; Movements; Voice and Speech.

This volume, like the preceding ones, is complete in itself, although constituting part of a general work on Physiology. The author has taken great care to present a full review of what is known concerning every subject embraced in his work. It is, consequently, a very valuable work, both for the student and practitioner.

The Journal of the Gynæcological Society of Boston. A Monthly Journal, devoted to the advancement of the knowledge of the Diseases of Women. Edited by Winslow Lewis, M.D., Horatio R. Storer, M.D., and Geo. H. Bixly, M.D., and published by James Campbell, 18 Tremont St., Boston. Pp. 387. Price, $2.50.

We have received from the publisher, the two first volumes of this Journal, elegantly bound, and lettered.

While we differ widely from the editors of the Gynæcological Journal, in regard to some questions of propriety, we give them credit for commendable efficiency in the conduct of their Journal, and freely commend it to our readers as containing a large amount of valuable matter in relation to the diseases peculiar to females. The two nicely bound volumes before us can be had of the publisher for $2.50 each.

---

Naval Hygiene. By Joseph Wilson, Surgeon United States Navy; with an appendix: Moving wounded men on Shipboard, by Albert C. Gorgas, Surgeon United States Navy. Reported to the Bureau of Medicine and Surgery. Published by order of the Navy Department. Washington, D.C. 1870.

We have received a neatly printed volume with the above title, from the Naval Medical Bureau, at Washington. It is an interesting work, and of special value to all who have anything to do with the shipping or naval interests.

---

Shaw's Medical and Surgical Case Book. E. P. Stevens & Co., Publishers, Chicago. 1870.

This is a substantially bound blank book, conveniently ruled and headed, for recording cases, either medical or surgical. There is also a department for inserting photographic plates, representing diseased conditions. It is one of the most convenient books for the simple purpose of keeping a record of cases that we have seen.

---

The Bromides: Their Physiological Effects and Therapeutic Uses. By Z. McElroy, M.D., Zanesville, Ohio.

This is a pamphlet of 26 pages, reprinted from the *New York Medical Journal*, of July, 1870. The author, during the past few years, has written many essays, or rather articles for different medical periodicals, all designed to sustain certain theories regarding what has been termed a *physical basis of life.* Following out the general train of thought prominently enunciated by Mr. Huxley, Dr. McElroy endeavors to trace all the phenomena of living beings in health and disease, to two processes, namely, nutrition or construction, and disintegration or waste. From the physical changes in matter involved in these processes, result all the forces and phenomena of life, mental and physical; and, furthermore, these changes are themselves the result of the operation of the same forces and laws that control inorganic matter. Like Mr. Huxley, he endeavors to discard the existence of any special *vital force* or property, and yet he only substitutes another term "*form force*," and gives to it very nearly the same attributes. Assuming that all the phenomena of living beings arise from the two processes, *nutrition* and disintegration, Dr. McElroy naturally infers that all remedial agents produce their effects by either promoting or retarding these processes.

The specific action of the Bromides, he regards as hastening the process of disintegration, and, consequently, applicable to the treatment of such cases only, as are accompanied by the retention of surplus material in one or more of the tissues of the body. He denies that they possess any direct hypnotic effect or power. We do not think the views of Dr. McElroy are sustained either by experiments or correct clinical observation. But we have neither time not space to pursue the subject further at present.

